# Morphology‐Controlled Aluminum‐Doped Zinc Oxide Nanofibers for Highly Sensitive NO_2_ Sensors with Full Recovery at Room Temperature

**DOI:** 10.1002/advs.201800816

**Published:** 2018-07-23

**Authors:** Amit Sanger, Sung Bum Kang, Myeong Hoon Jeong, Min Ji Im, In Young Choi, Chan Ul Kim, Hyungmin Lee, Yeong Min Kwon, Jeong Min Baik, Ho Won Jang, Kyoung Jin Choi

**Affiliations:** ^1^ School of Materials Science and Engineering KIST‐UNIST Ulsan Center for Convergent Materials (KUUC) Ulsan National Institute of Science and Technology (UNIST) Ulsan 44919 Republic of Korea; ^2^ Department of Materials Science and Engineering Research Institute of Advanced Materials Seoul National University Seoul 08826 Republic of Korea

**Keywords:** collision frequencies, fiber alignment, finite‐difference time‐domain simulations, free‐standing nanofibers, room‐temperature gas sensors

## Abstract

Room‐temperature (RT) gas sensitivity of morphology‐controlled free‐standing hollow aluminum‐doped zinc oxide (AZO) nanofibers for NO_2_ gas sensors is presented. The free‐standing hollow nanofibers are fabricated using a polyvinylpyrrolidone fiber template electrospun on a copper electrode frame followed by radio‐frequency sputtering of an AZO thin overlayer and heat treatment at 400 °C to burn off the polymer template. The thickness of the AZO layer is controlled by the deposition time. The gas sensor based on the hollow nanofibers demonstrates fully recoverable n‐type RT sensing of low concentrations of NO_2_ (0.5 ppm). A gas sensor fabricated with Al_2_O_3_‐filled AZO nanofibers exhibits no gas sensitivity below 75 °C. The gas sensitivity of a sensor is determined by the density of molecules above the minimum energy for adsorption, collision frequency of gas molecules with the surface, and available adsorption sites. Based on finite‐difference time‐domain simulations, the RT sensitivity of hollow nanofiber sensors is ascribed to the ten times higher collision frequency of NO_2_ molecules confined inside the fiber compared to the outer surface, as well as twice the surface area of hollow nanofibers compared to the filled ones. This approach might lead to the realization of RT sensitive gas sensors with 1D nanostructures.

## 1 Introduction

We are witnessing the beginning of the fourth industrial revolution represented by technological innovations, such as internet of things (IoT), robotics, artificial intelligence, and quantum computing, which will fundamentally change the human society and lifestyles.^1^ IoT is the ecosystem of physical objects such as devices, home appliances, vehicles, and even animals or people embedded with sensors, electronics, software, actuators, etc., connected through the internet.^2^ Gas sensors that are linked to IoT are recognized as key technological devices that can maximize the potential of IoT by detecting the leakage of hazardous gases and alerting the consumers in real time.^3^ Unfortunately, most gas sensors operate at high temperatures, ≈300 °C, because the gas molecules are not reactive to the sensing materials at room temperature (RT). The high‐temperature operation of these gas sensors increases the power consumption and causes frequent battery replacement, which can limit the utilization of IoT sensors to their fullest potential.^4^

Recently, several types of gas sensors that operate at RT have been developed based on transitional metal dichalcogenides (TMDs), graphene, and carbon nanotubes (CNTs).^5^, ^6^, ^7^, ^8^, ^9^, ^10^, ^11^, ^12^, ^13^ Cho et al. studied the RT sensing properties of an atomic layer of MoS_2_ to NO_2_, owing to the availability of many active edge sites and extraordinary high carrier mobility.^14^, ^15^ Jeong et al. prepared CNT/reduced graphene hybrid films exhibiting RT NO_2_‐gas‐sensing properties attributed to the high specific area of the hybrid composite film.^16^ Yuan et al. prepared chemically modified graphene and demonstrated its RT sensitivity to NO_2_ gas, which is attributed to its high carrier mobility and large specific surface.^17^ However, the practical application of these gas sensors is impeded by some fundamental issues. Kim et al. reported that TMD‐based gas sensors encounter several drawbacks such as poor gas selectivity, short lifetimes, sluggish recovery characteristics, and difficulty in the fabrication of large‐scale devices.^18^ Zaporotskova et al. reported that CNT‐based gas sensors exhibit high sensitivity but require a long exposure time, irreversible changes in CNT conductivity, lack of selectivity, and inability to identify gases with low adsorption energies.^19^ Varghese et al. reported that graphene gas sensors have drawbacks in terms of their sensitivity, detection limit, and repeatability.^20^ Even though the above‐noted sensors are claimed to have RT sensitivity, their responses have been very unreliable with incomplete recovery. Hence, these sensors are not suitable for practical applications.

Semiconductor metal oxide (SMO)‐based chemiresistive gas sensors are promising devices that can overcome these issues owing to their high response to various gases, low cost, easy synthesis methods, and long life times.^21^, ^22^ Current research efforts are directed toward developing high‐performance gas sensors based on 1D SMO nanostructured materials (e.g., nanowires, nanobelts, nanorods, and nanotubes) with RT operation and low fabrication costs, to establish nanostructure–sensing property correlations.^23^ Recently, Shankar and Rayappan prepared ZnO nanorods with flat, sharp pencil‐type, and blunt‐end morphologies and demonstrated their RT sensitivity to ethanol.^24^ Jiang et al. studied the RT sensing properties of hollow SnO_2_ nanocrystalline tubes to NO*_x_*.^25^ Zhang et al. prepared dendritic ZnO nanostructures that exhibit RT H_2_S‐sensing properties.^26^ In these cases, the mechanism underlying the reported RT gas‐sensing properties are not clearly explained and the characteristics are attributed to the complicated, fascinating, and unusual nanostructure and it requires further study.

Herein, inspired by the suspension bridge method, we describe the synthesis of free‐standing hollow aluminum‐doped zinc oxide (AZO) fibers on a custom‐made copper electrode frame. The resulting fibers were characterized by standard techniques. For comparison, hollow and filled AZO fiber structures were tested for sensing a low concentration of the NO_2_ gas. In this study, hollow fibers displayed fully recoverable RT sensitivity. Based on the finite‐difference time‐domain (FDTD) simulation, we propose that the RT gas response is due to the high collision frequency of NO_2_ molecules inside the hollow core. The approach described in this work may lead to the realization of more RT‐sensitive gas sensors based on 1D nanostructures.

## 2 Results

**Figure 1:**
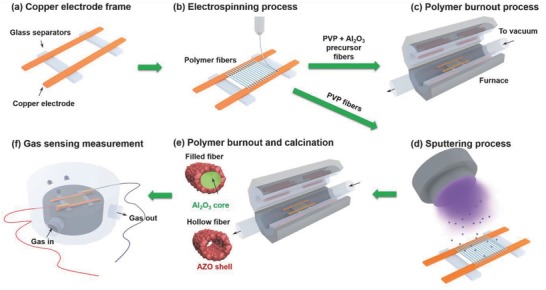
Schematic illustration of the overall fabrication process of the hollow/filled AZO fibers: a) Fabrication of a custom‐made copper frame, b) electrospinning of PVP and PVP‐Al_2_O_3_ precursor fibers, c) polymer burnout process of the Al_2_O_3_ precursor fiber, d) sputtering deposition of an AZO layer on the PVP and Al_2_O_3_ fibers, and e) polymer burnout and calcination of AZO‐coated fibers. f) Gas detection process using the final prototype device.

**Figure 2:**
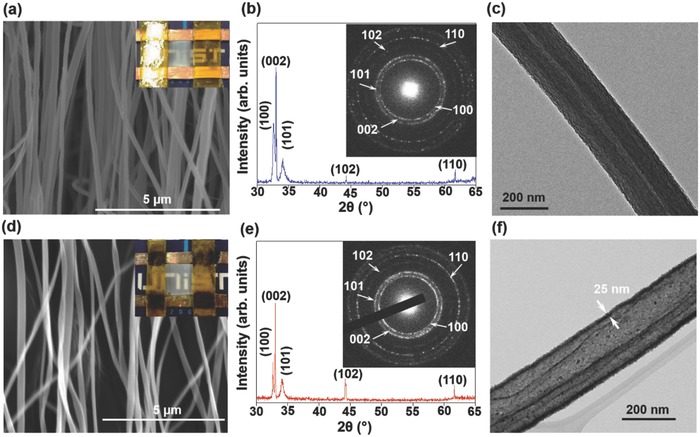
a–c) SEM image, XRD spectrum, and TEM image, respectively, the free‐standing filled AZO fiber and d–f) free‐standing hollow AZO fiber. Insets in (a) and (d) show the corresponding optical micrographs and insets in (c) and (f) show the corresponding SAED patterns.

A schematic of the overall fabrication process of the hollow/filled AZO fibers is shown in **Figure** **1**. The free‐standing nanofibers are fabricated using polyvinylpyrrolidone (PVP) and Al_2_O_3_‐PVP precursor fiber template electrospun on a copper electrode frame followed by radio‐frequency sputtering of an AZO thin overlayer and heat treatment at 400 °C to burn off the polymer template. **Figure** **2**a,d shows the scanning electron microscopy (SEM) images of the filled and hollow AZO fibers and the corresponding optical images of the prototype devices, showing the aligned fibers over the entire length, respectively. The average diameter of the fibers is ≈200 nm. As shown in Figure 2b,e, the filled and hollow AZO fibers exhibit almost the same diffraction patterns with five distinct peaks corresponding to (100), (002), (101), (102), and (110) planes of polycrystalline wurtzite phase (JCPDS No. 361451). The corresponding selected area electron diffraction (SAED) patterns of the filled and hollow fiber are shown in the inset of Figure 2b,e, respectively. These SAED patterns show similar and clearly visible rings with distinct spots, indicating the polycrystalline nature of these fibers. Not a single diffraction spot in the SAED pattern or peak in the XRD pattern corresponding to Al_2_O_3_ is observed for the filled fibers. For clarification, the transmission electron microscopy (TEM) image and the corresponding diffuse SAED pattern of Al_2_O_3_ were also obtained and are shown in Figure S1a,b (Supporting Information), indicating the amorphous nature of the core material. The TEM images in Figure 2c,f show typical, uniform, filled, and hollow 1D fibers, respectively. The thickness of the deposited AZO layer on these fibers is ≈25 nm. Figure S2a (Supporting Information) shows the transmittance spectrum of the filled and hollow AZO fibers in the wavelength range of 300–800 nm. The transmittance of the hollow fibers is close to 90% in the visible range, whereas that of the filled fibers is close to 80%. The decrease in the transmittance might be due to the presence of Al_2_O_3_ core in the filled fibers. At lower wavelengths, the zero transmission (UV off) behavior is observed, which can be attributed to the Burstein–Moss effect.^27^ According to this phenomenon, in the presence of UV light, the Fermi level of the AZO layer penetrates the conduction band and overly populates it with the electron carriers, leading to a sharp decrease in the transmittance.

The fabricated nanofibers are well connected to the copper electrodes, resulting in the formation of ohmic contacts (Figure S2b, Supporting Information). The doped ZnO fibers have a conductivity nearly twice that of undoped ZnO. For comparison, the gas‐sensing behavior of Al‐doped and undoped ZnO toward NO_2_ at 250 °C was examined and the results are shown in Figure S3 (Supporting Information). The AZO fibers show three times higher sensitivity than the undoped ZnO fibers. It is well accepted that the gas‐sensing mechanism of an SMO gas sensor depends on the gas–material interaction involving the targeted energetic gas molecules and the surface area of the SMO layer.^23^, ^28^, ^29^ The surface‐adsorbed oxygen molecules (from air) pull electrons, leading to the formation of a potential barrier that is sensitive to the electron carrier concentration in the SMO layer. When an SMO (n‐type) is exposed to the oxidizing gas, NO_2_, it extracts electrons from the surface resulting in further decrease in the current and increase in the potential barrier height^30^ (Supporting Information). The same mechanism may be applied to the AZO nanofiber. Here, the doped Al atoms replace the Zn atoms in the ZnO lattice structure and increase the carrier concentration.^31^ A high carrier concentration (*N*_d_) might lead to a low response owing to a small change in the depletion width (Supporting Information). However, the doped substitutes, Al atoms, act as the preferential available active adsorption sites for the surface‐adsorbed oxygen molecules, favoring higher surface reactivity of the Al‐doped ZnO sensing layer over undoped ZnO to NO_2_ under the same experimental conditions.^32^ Considering the results, we finally selected AZO as the active material to design our gas sensors over ZnO.

**Figure 3:**
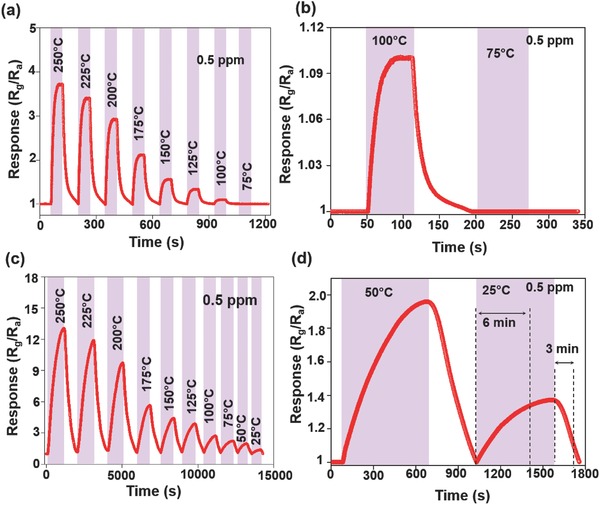
a) Sensing response of the free‐standing AZO filled nanofiber gas sensor to 0.5 ppm NO_2_ versus operating temperature; b) the response and recovery behaviors of the free‐standing AZO‐filled nanofiber gas sensor to 0.5 ppm NO_2_ at 100 and 75 °C; c) sensing response of the free‐standing AZO hollow nanofiber gas sensor to 0.5 ppm NO_2_ versus operating temperature; and d) the response and recovery behaviors of the free‐standing AZO hollow nanofiber gas sensor to 0.5 ppm NO_2_ gas at 50 °C and room temperature.

Representative resistance response of the filled AZO fibers exposed to 0.5 ppm NO_2_ as a function of temperature is plotted in **Figure** **3**a. The filled fibers show a pronounced increase in their response at high temperatures and they become unresponsive below 75 °C (Figure 3b). The response behavior of hollow AZO nanofibers during cyclic exposures to 0.5 ppm NO_2_ gas as a function of temperature is shown in Figure 3c. In contrast, the hollow fibers exhibit remarkable RT sensitivity and three times higher sensitivity than the filled fibers for 0.5 ppm NO_2_ gas. The RT response behavior is fully recovered in 3 min with a response time of 6 min (Figure 3d). The response behaviors of filled and hollow AZO nanofiber gas sensors to various concentrations (10–0.5 ppm) of NO_2_ at 250 °C are shown in Figure S4 (Supporting Information). Owing to experimental limitations, the gas concentration was maintained between 0.5 and 10 ppm while the temperature was maintained below 250 °C. Higher gas responses are observed for higher concentrations of NO_2_. Here, hollow fibers show three times higher sensitivity toward 0.5 ppm NO_2_ gas than filled fibers. However, the observed response and recovery times are found to be 11 and 10 min, much slower than those of the filled fibers, 20 and 30 s, at 250 °C, respectively. Figure S5a,b (Supporting Information) shows the responses of filled and hollow AZO fibers as a function of the concentration of NO_2_ at 250 °C, indicating a typical power law dependence. Whereas the calculated slope for hollow AZO fibers is 0.49 and the lowest detection limit (LDL) is ≈0.2 ppm, the calculated slope and LDL for filled AZO fibers are higher at 0.39 and ≈0.4 ppm, respectively. The calculation of LDL is discussed in the Supporting Information. Compared to other previously reported gas sensors based on ZnO nanocrystals, Sn and In‐doped ZnO thin films, or ZnO nanowires, our gas sensor shows notable gas response at RT as well as at high temperature^33^, ^34^, ^35^, ^36^ (Figure S6, Supporting Information).

**Figure 4:**
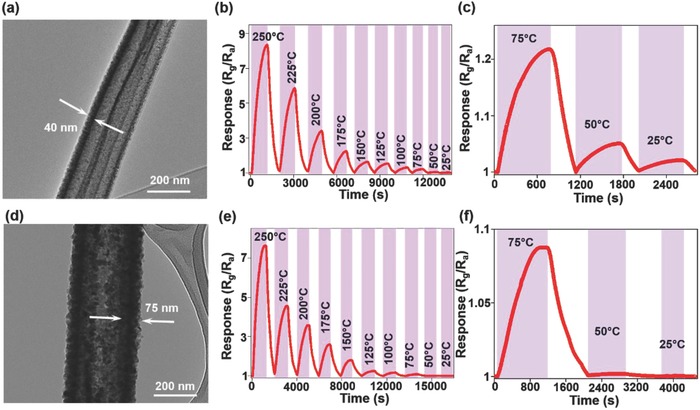
TEM image, sensing response to 0.5 ppm NO_2_ versus operating temperature, and the response and recovery behaviors, respectively, of a gas sensor based on a–c) 40 nm thick free‐standing hollow AZO nanofibers and d–f) 75 nm thick free‐standing hollow AZO nanofibers.

Two major distinctive characteristics, viz., (i) slow response–recovery times and (ii) high and RT sensitivity were observed in the case of sensing by the hollow fibers in comparison to those of the filled fibers. The slow response–recovery times of NO_2_ gas sensing by hollow nanofibers can be explained by the diffusion kinetics and penetration depth of the gas molecules. First, the NO_2_ gas molecules are adsorbed on the AZO surface, followed by their diffusion through the AZO layer and eventual trapping inside the hollow core. To support our assumption, we fabricated hollow fibers of varying thicknesses ranging from 40 to 75 nm and evaluated their sensing characteristics. The fiber thickness was controlled by varying the deposition time of sputtering, with all other parameters kept constant (**Figure** **4**). The gas‐response curves of the nanofibers with 40 nm thickness show a trend similar to that of the previous one (25 nm) with a response time of 8 min at RT. The results suggest that the gas requires a longer time to diffuse through the thicker wall of the AZO layer, owing to the large penetration depth. In the case of filled fiber, the adsorption of the gas molecules takes place only on the active adsorption sites of the outer surface of the AZO layer as there are no available adsorption sites at the AZO/Al_2_O_3_ interface. Hence, the gas molecules cannot diffuse through the AZO layer of the filled fiber, leading to fast response–recovery process of the gas sensor.

However, hollow nanofibers have a three times higher gas sensitivity for 0.5 ppm NO_2_ at 250 °C with significant RT gas response than the filled fibers. Considering the fiber morphology, hollow fibers have a nearly two times higher surface area than the filled fibers. Hence, there are also certain other deciding factors responsible for the high and RT sensitivity of hollow fibers, which needs to be investigated. As the thickness of the hollow fiber increases, the sensitivity starts to decrease and the 75 nm thick hollow nanofiber is almost insensitive to the gas at RT. This is because the depletion width remains same under the same experimental conditions but the equiaxed nanograins change to elongated nanograins and the undepleted region becomes a significant part of the fiber (Equations (S11)–(S13), Supporting Information). The increase in the undepleted region with the fiber thickness leads to a decrease in the sensitivity.

The selectivity of AZO fibers gas sensor with respect to potentially interfering available gases of 0.5 ppm NO_2_, 100 ppm hydrogen (H_2_), and 50 ppm carbon monoxide (CO) was investigated at 250 °C (Figure S7a, Supporting Information). The sensor showed a remarkably high gas response (*R*_g_/*R*_a_ ≈ 13 and 3.7) for NO_2_ gas with a weak response (*R*_a_/*R*_g_ < 2.7 and 1.3) toward the other gases for hollow and filled AZO fibers, respectively, confirming the high selectivity toward NO_2_. The humidity effect on the gas sensor response is investigated with different relative humidity (RH) levels at RT (Figure S7b, Supporting Information). Water vapor adsorbed onto the AZO sensing layer act as a reducing gas, mitigating the sensor response to NO_2_.^37^ The sensor response reduced only 3% under 75% RH condition, warranting the real field application of these sensors. The cyclic stability and response retention of hollow AZO fiber is tested with 0.5 ppm NO_2_ at RT (Figure S7c,d, Supporting Information). The cyclic behavior shows stable gas response over 30 cycles. The response retention exhibits constant sensor signals (96.3% change) for 60 d, indicating long‐term stability of the sensor.

To assess the RT gas‐sensing mechanism, we carried out a series of experiments based on X‐ray photoelectron spectroscopy (XPS) and simulation studies. XPS analysis was performed for pure AZO thin film and AZO fibers to examine the role of unintentional carbon doping or catalysts that might initiate the RT sensitivity (Figure S8, Supporting Information). The O1s XPS spectra of pure AZO thin film and hollow fibers are shown in Figure S8a,e (Supporting Information). Both the XPS spectra show a peak at 530 eV attributed to O^2−^ of the Zn—O bonds and another peak at 531 eV attributed to the surface‐absorbed oxygen species such as OH− and carbonates.^38^ The peak at 532 eV in the XPS spectrum of AZO hollow fibers is attributed to the Zn—O—C bonds.^39^ The C1s spectrum of pure AZO thin film shows a peak centered at 284 eV assigned to the impurity‐induced graphitic sites (Figure S8b, Supporting Information). In contrast, the C1s spectrum of AZO fibers could be deconvoluted into four components at 283, 284, 286, and 289 eV (Figure S8f, Supporting Information). The peak centered at 284 eV is assigned to the residual carbon and pure graphitic sites in the nanostructures. The peak at 289 eV is attributed to the adsorption of CO_2_ and structural carbonate species comprising C=O.^40^ The peak at 286 eV could be due to the Zn—O—C bonds, whereas the peak at 283 eV could be assigned to Zn—C bonds connected to oxygen vacancies because additional electron density would be imposed by the negatively charged oxygen vacancies on carbon in Zn—C—O bond.^41^ The presence of Zn—C bonding changes the energy level and affects the adsorption band edge and band gap.^39^ The Zn2p (Figure S8c,g, Supporting Information) and Al2p (Figure S8d,h, Supporting Information) spectra are found to be similar for the thin film and hollow fibers.^42^ In the Al2p spectra, the peak at ≈74 eV suggests that Al atoms substitute the Zn atoms in the AZO layer and act as Al donors and the doping level corresponds to that in the sputtering target.^43^ Hence, the presence of Zn—C might have led to the overall increment in the gas‐sensing performance of hollow fibers, as shown in Figure S9 (Supporting Information). However, the XPS results of hollow and filled fibers indicate almost the same carbon content and well corroborate with the obtained energy dispersive X‐ray spectroscopy (EDS) measurements (Figures S10 and S11, Supporting Information), discounting the possible role of carbon doping in the RT sensitivity. Although carbon doping might enhance the overall sensitivity of the fibers, it is not directly related to the RT sensitivity of hollow nanofibers, and the underlying mechanism remains unclear.

**Figure 5:**
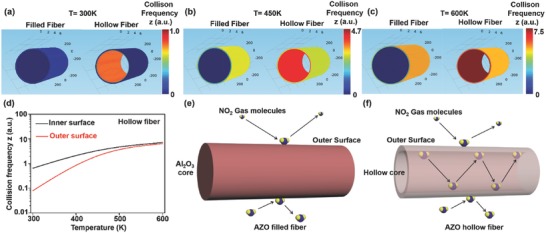
Simulated collision frequency data for hollow and filled fibers at a) 300, b) 450, and c) 600 K, respectively. d) Collision frequency versus temperature for the outer and inner surfaces of the hollow fibers. Schematic showing the interaction of the gas molecules with e) the outer surface of a filled fiber and f) the inner and outer surfaces of a hollow fiber.

**Figure 6:**
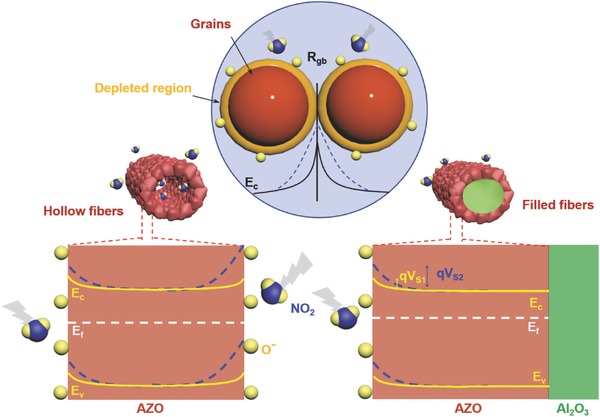
Schematic illustration of the NO_2_ gas‐sensing mechanism of hollow and filled AZO fibers.

Therefore, to explain the RT gas‐sensing mechanism, the Maxwell–Boltzmann distribution kinetic theory is applied.^44^ During the gas–material interaction, the gas adsorption rate is mainly proportional to three factors: The energetic gas molecules, available active sites, and “collision frequency.” The energy of gas molecules can be controlled by the temperature. At high temperatures, the energetic molecules can easily adsorb on the surface. At low temperatures, the energy of the gas molecules is too low to react with the active sites. The number of available active sites is directly dependent on the high specific surface area. When the concentration of gas molecules is too high, there is a significant competition between the gas molecules to adsorb on the available active sites. At low concentrations, the active sites become easily available. The “collision frequency” depends on the temperature as well as the shape confinement and can be expressed as the average rate at which two reactants collide in a defined system. Therefore, the collision frequency is the only remaining factor that can determine the RT sensitivity. Considering the above facts, we used an FDTD simulation to simulate the active area field distribution during the gas sensing by the nanofibers (**Figure** **5** and Figures S12 and S13, Supporting Information). In the simulation, the hollow and filled fiber under the laminar flow of gas molecules in a defined space is considered. Periodic boundary conditions in the *x* and *y*‐directions and a perfectly matched layer in the *z*‐direction are applied. At high temperatures of 450 and 600 K, FDTD simulation results reveal that the collision frequency is almost two times higher inside the core of the hollow AZO fibers than on the outer surface (Figure 5d). Even at RT, 25 °C (≈300 K), owing to the confinement effect, the collision frequency is comparatively almost eight times higher inside the core of the hollow AZO fibers than on the outer surface. The reason behind the low collision frequency on the outer surface is the longer mean free path and larger available free space of the fiber. As the molecules are trapped inside the hollow core, the collision frequency becomes very high owing to their constrained motion, short mean free path, and low degree of freedom. This sharp difference in the collision frequency also changes the potential barrier height (**Figure** **6** and the Supporting Information). Hence, this continuous encounter between the gas molecules inside the core leads to higher gas sensitivity at RT compared to that of the filled fibers. This is the first time that a clear mechanism behind the RT gas sensitivity of hollow nanofibers is proposed.

## 3 Conclusion

Inspired by the suspension bridge method, we describe the synthesis of free‐standing hollow AZO fibers on a custom‐made copper electrode frame. The high surface‐to‐volume ratio of the free‐standing hollow AZO fibers renders it an excellent gas‐sensing material with fully recoverable and high RT sensitivity toward 0.5 ppm NO_2_ gas. A comparative study of the gas‐sensing properties of hollow and filled AZO fiber structures was performed both experimentally and theoretically. An FDTD simulation reveals that the RT gas response is due to the high collision frequency of NO_2_ molecules inside the hollow core. We speculate that the proposed approach and mechanism will lead to the realization of RT sensitive gas sensors based on 1D nanostructures.

## 4 Experimental Section

*Fabrication of the Copper Electrode Frame*: To explore the impact of NO_2_ gas on the carrier transport inside the nanofibers, a simple yet robust sensor was fabricated by electrospinning the nanofibers on a custom‐made copper electrode frame. The copper electrode frame was prepared with copper legs separated by glass separators using a high‐temperature adhesive. The area gap between the copper legs kept constant at ≈1 cm^2^. The copper legs act as the electrodes and the glass separators between the electrodes provide electrical insulation and facilitate the suspension of the electrospun nanofibers. The copper electrode frame enables the direct electrospinning of free‐standing nanofibers on the electrodes instead of the conventional complicated template formation and transfer process, rendering the device fabrication very easy. Moreover, free‐standing nanofibers provide additional advantages such as faster gas permeability and optical transparency upon controlling the density of nanofibers or the electrospinning time.

*Synthesis of the Hollow Fiber Network*: A solution of PVP (Sigma‐Aldrich) was prepared by dissolving PVP (1.6 g, *M*_w_ = 1 300 000 g mol^−1^) powder in ethanol (25 mL, Samchun Chemicals Korea) under vigorous stirring at 750 rpm and 40 °C for 1 h. For electrospinning, PVP solution (6 mL) was placed in a 10 mL plastic syringe tipped with a 26 gauge stainless steel needle. A high voltage of 9.5 kV was applied to the metal needle with the copper electrode frame grounded.^45^, ^46^ The PVP solution was injected into the needle at a constant rate of 0.8 mL h^−1^ with a syringe pump. Electrospinning was carried out at a needle‐to‐frame distance of 15 cm. After electrospinning, a layer of AZO (2 wt%, 99.999% pure, ITASCO) was deposited on the PVP fiber template using an radio‐frequency (RF) power of 50 W under a flow of Ar (20 sccm) at a working pressure of 5 mTorr and RT to prevent the thermal decomposition of the PVP template. Afterward, the samples were heated in a furnace at 400 °C for 1 h to burn the polymer template and crystallize the AZO overlayers.

*Synthesis of the Filled Fiber Network*: High‐purity aluminum 2,4‐pentanedionate [Al(CH_3_COCHCOCH_3_)_3_] (Sigma‐Aldrich, purity 99.99%) was used as the precursor for the synthesis of Al_2_O_3_ nanofibers. First, a solution of the alumina precursor was prepared by dissolving aluminum 2,4‐pentanedionate (0.3 g) in acetone (5 mL, Samchun Chemicals Korea) under constant and vigorous stirring. Then, a solution of PVP was prepared by dissolving PVP powder (0.3 g) in ethanol (5 mL) under constant and vigorous stirring. Both solutions were mixed and stirred vigorously to obtain a clear transparent solution for electrospinning. For electrospinning, the same procedure employed for the electrospinning of hollow fibers was used. Then fibers were heated in a furnace at 400 °C for 1 h to remove the PVP polymer and other organic components by thermal decomposition, thereby transforming the Al_2_O_3_ precursor nanofibers into Al_2_O_3_ core nanofibers. Then, a similar sputtering process as explained in the previous section was followed to deposit an AZO layer. The samples were heated again at 400 °C for 1 h to crystallize the AZO layer. The entire synthesis process is graphically represented in Figure 1.

*Characterization*: The morphology of the fibers was examined by field emission SEM (FEI Quanta 200FEG). Phase identification was performed by X‐ray diffraction using a Bruker D8 Advance diffractometer with Cu K_α_ radiation, and the microstructure was observed by TEM using a JEOL JEM‐2100F microscope. TEM sample preparation involved the transfer of the free‐standing fibers onto a carbon‐coated copper grid. XPS (Thermofisher) was performed with an Mg K_α_ X‐ray source. Optical transmission spectra were recorded using a UV–vis spectrometer (Cary Varian).

*Gas‐Sensing Measurements*: Gas‐sensing properties were evaluated in a stainless‐steel probe chamber (≈125 cm^3^) equipped with a Keithley 2636A electrometer system, a temperature‐control system (RT to 250 °C), and a mass flow controller (MS Tech, Korea) by monitoring the changes in the DC current under a constant applied voltage (1.0 V) while varying the concentration of NO_2_ in dry N_2_ at atmospheric pressure without any vacuum precondition. N_2_ is often used as carrier gas to study the chemical kinetics of the interaction between a test gas and the metal oxide surface due to chemically non‐reactive nature. The sensor was placed on a heater with an electrically insulating polyimide sheet to prevent short circuit. Subsequently, before gas exposure for testing, the gas sensors were stabilized in dry N_2_ ambient at the test temperature. Then, various concentrations of NO_2_ gas (0.5–10 ppm) diluted with N_2_ were exposed to the gas sensors. After exposure to a specific concentration, the system was flushed with N_2_ gas to restore the sensor to its original current level and the measurement at a different concentration was resumed. The results reported in this report were obtained from a long series of experiments on one of the devices. The sensor response is defined as *S = (R*_g_ */R*_a_), where, *R*_g_ is the resistance measured in the presence of the target gas mixed with N_2_ stream, and *R*_a_ is the resistance measured with only the N_2_ stream.

*FDTD Simulation on Hollow and Filled AZO Nanofibers*: The gas‐sensing mechanism of the SMO‐based gas sensor involves the adsorption of the target gas molecules on the sensing material surface and a subsequent series of chemical reactions between the adsorbed molecules and charge carriers in the sensing materials. The gas adsorption rate is mainly proportional to three factors: the concentration of the energetic gas molecules, available active sites, and “collision frequency.” The concentration of the energetic gas molecules can be determined by the well‐known Maxwell–Boltzmann distribution kinetic theory and it depends on the average speed of gaseous molecules at a given temperature. The movement of the gas molecules is interrupted by collisions with a physical boundary such as the surface of the nanofibers. The number of collisions per unit time, known as the collision frequency, can be calculated from the momentum and velocity distribution of gas molecules (details are provided in the Supporting Information). In this study, COMSOL Multiphysics modeling software equipped with a particle tracing module was used to solve the Maxwell–Boltzmann distribution kinetics equations. In this simulation, the gas flow is considered laminar, and the grain size of AZO is assumed to be 5 nm. The wall thickness is considered ≈25 nm, comparable to the AZO layer thickness, as determined by TEM. The boundary conditions of the gas molecules with walls are described elsewhere,^47^, ^48^, ^49^ with details provided in the Supporting Information.

## Conflict of Interest

The authors declare no conflict of interest.

## Supporting information

Supplementary
