# Supplementary material for: Morphology‐Controlled Aluminum‐Doped Zinc Oxide Nanofibers for Highly Sensitive NO2 Sensors with Full Recovery at Room Temperature
Source: Adv Sci (Weinh). 2018 Jul 23;5(9):1800816. doi: 10.1002/advs.201800816 (PMC6145242; doi:10.1002/advs.201800816)
Supplement: Supplementary file 1 — Supplementary [file ADVS-5-1800816-s001.pdf]

Copyright WILEY-VCH Verlag GmbH & Co. KGaA, 69469 Weinheim, Germany, 2018.

ADVANCED SCIENCE
Open Access

Supporting Information

for *Adv. Sci.,* DOI: 10.1002/advs.201800816

Morphology-Controlled Aluminum-Doped Zinc Oxide Nanofibers for Highly Sensitive NO_2 Sensors with Full Recovery at Room Temperature

*Amit Sanger, Sung B. Kang, Myeong H. Jeong, Min J. Im, In Y. Choi, Chan U. Kim, Hyungmin Lee, Yeong M. Kwon, Jeong M. Baik, Ho W. Jang, and Kyoung J. Choi**

Copyright WILEY-VCH Verlag GmbH & Co. KGaA, 69469 Weinheim, Germany, 2016.

Supporting Information

Morphology-Controlled Aluminum-Doped Zinc Oxide Nanofibers for Highly Sensitive NO_2 Sensors with Full Recovery at Room Temperature

*Amit Sanger, Sung B. Kang, Myeong H. Jeong, Min J. Im, In Y. Choi, Chan U. Kim, Hyungmin Lee, Yeong M. Kwon, Jeong M. Baik, Ho W. Jang, Kyoung J. Choi**

E-mail: choi@unist.ac.kr

1. Calculation of the lowest detection limit

Lowest detection limit (LDL) can be calculated from linear fit data of sensor response versus gas concentration curve.

$$LDL = \frac{3.3\ x\ standrad\ deviation\ in\ intercept}{slope} \quad \text{(S1)}$$

Standard deviation in intercept

$$b = a\sqrt{n} \quad \text{(S2)}$$

where, a is the standard error of the intercept and n is the total calculated points.

2. Gas-sensing mechanism

The gas-sensing mechanism of the aluminum-doped zinc oxide (AZO) fiber sensor is explained as follows: Initially, oxygen molecules (from air) adsorb on the fiber surface and pull electrons, leading to the formation of a potential barrier (qV_{s1}) that is sensitive to the charge carrier concentration N_d[1]. The type of adsorbed oxygen species depends on the temperature. As shown in Figure 4 and 6 (main manuscript), an increase in the height of qV_{s1} to qV_{s2} under exposure to NO_2 leads to further decrease in the current.

$$O_{2(air)} \rightarrow O_{2(adsorbed)}\ , \quad \text{(S3)}$$

$$O_{2(adsorbed)} + e^- \rightarrow O^-_{2(adsorbed)}, \quad \text{(S4)}$$

$$O^-_{2(adsorbed)} + e^- \rightarrow 2O^-_{(adsorbed)}, \text{ and} \quad \text{(S5)}$$

$$NO_2 + 2O^-_{(adsorbed)} \rightarrow NO^-_2 + O^-_{2(adsorbed)} \quad \text{(S6)}$$

The gas response based on the potential barrier height can be represented by Equations S7 and S8[2]:

$$V_{s1} = \frac{2\pi Q_s^2}{\varepsilon N_d} = \frac{2\pi (qN_s)^2}{\varepsilon N_d} \text{ and} \quad \text{(S7)}$$

$$Response = \frac{R_g}{R_a} \approx exp\left(\frac{e(V_{s2} - V_{s1})}{kT}\right), \quad \text{(S8)}$$

where e is the elementary charge, Q_s is the surface charge density, k is the Boltzmann constant, T is the operating temperature, and ε is the dielectric constant of AZO. The introduction of free-standing fibers to replace the thin film leads to the two-fold geometrical increment in the surface area as well as higher sensitivity. Here, the high sensitivity may be associated to the high permeability of the free-standing fiber networks to the gas molecules, which facilitates the diffusion of gaseous molecules. Assuming a coaxial geometry of the AZO nanofibers, the conductance under exposure to air (G_{air}) and NO_2 (G_{gas}) can be represented as follows[3]:

$$G_{air} = qN_D \mu_n \frac{\pi(D-2L_{air})^2}{4l} \text{ and} \qquad \text{(S9)}$$

$$G_{gas} = qN_D \mu_n \frac{\pi(D-2L_{gas})^2}{4l}, \qquad \text{(S10)}$$

where μ_n is the electron mobility, D is the thickness of the AZO layer, l is the fiber length, and L_{air} and L_{gas} are the electron depletion widths in air and NO_2, respectively. The electron depletion width can be represented as follows:

$$L = \lambda_D \left(\frac{qV_s}{kT}\right)^{1/2} \text{ and} \qquad \text{(S11)}$$

$$\lambda_D = \left(\frac{\varepsilon kT}{2\pi e^2 N_d}\right)^{1/2}, \qquad \text{(S12)}$$

where, λ_D is the Debye length. Thus, the conductivity of the fibers is mainly dependent on the variation in the electron depletion width and V_s. For L_{air} being equivalent to D/2, a higher variation in the conductance of fibers can be achieved, i.e., the surface-to-volume ratio has a major role on the transport properties. This describes the higher sensing performances of distinct structures with lower thicknesses. By changing the V_{s1} value from 0.1 to 3 V, the L_{air} for AZO was estimated to be ~20–50 nm using the relationship[4-6], $(2\varepsilon V_s/eN_d)^{1/2}$. Therefore, if L_{gas} is comparable to the thickness of the fibers, the gas response can be represented by Equation S13:

$$Response = \frac{R_g}{R_a} = \left(\frac{D-2L_{air}}{D}\right)^2 = \left(\frac{D-2\lambda_D\left(\frac{qV_{s1}}{kT}\right)^{1/2}}{D}\right)^2 \qquad \text{(S13)}$$

Therefore, compared to the thin film, the free-standing fiber network provides a higher surface area of the active material so that the effect of the potential barrier height and interaction with the target gas molecules leads to higher sensitivity. Thus, the higher NO_2-sensing properties can be justified by the transfer of electrons from the AZO fibers to the NO_2 molecules following their adsorption on the surface. Hence, in the presence of air, oxygen molecules are adsorbed on the surface of the AZO fibers, establishing an electron depletion region with a width, L_{air}. Under exposure to NO_2, the electrons transferred from the oxygen adsorbed on the AZO surface to the NO_2 molecules increase the electron depletion width, L_{gas}. Hence, the structure of the free-standing fibers leads to higher modulation in L than that of the thin films, giving rise to a higher gas response.

3. Finite-difference time-domain (FDTD) calculations

Maxwell–Boltzmann velocity distribution

$$< v >= \int_0^{\infty} V(\frac{m}{2\pi k_B T})^{0.5}\ e^{-\frac{m v_x{}^2}{2k_B T}} dv_x$$

$$< v > = A\Delta t \int_0^{\infty} v_x(\frac{m}{2\pi k_B T})^{0.5}\ e^{-\frac{m v_x{}^2}{2k_B T}} dv_x$$

$$< v > = A\Delta t \left(\frac{m}{2\pi k_B T}\right)^{0.5} \left(\frac{1}{2}\frac{2k_B T}{m}\right)$$

$$< v > = A\Delta t \left(\frac{k_B T}{2\pi m}\right)^{0.5} \qquad \text{(S14)}$$

Momentum distribution

$$dP_X = (2m_X)\left(\frac{1}{2}\frac{N}{V}(v_x{}^2 dt)A\right)$$

$$dP_X = \mathrm{m} v_x{}^2 \frac{N}{V} A dt$$

$$\frac{dP_X}{dt} = \mathrm{m} v_x{}^2 \frac{N}{V} A \qquad \text{(S15)}$$

Collision frequency

$$z = \frac{dP_X}{dt} < v > = \frac{N}{3V} m {v_x}^2 A^2 \Delta t \left(\frac{k_B T}{2\pi m}\right)^{0.5}$$

$$z = \frac{2}{3}\frac{U}{V} A^2 \Delta t \left(\frac{k_B T}{2\pi m}\right)^{0.5}$$

$$\propto \frac{2}{3V} A^2 \Delta t \left(\frac{k_B T}{2\pi m}\right)^{0.5} \qquad \text{(S16)}$$

A is the surface area, *Δt* is the time, *P* is the momentum, k_B is the Boltzmann constant, *T* is the temperature, *m* is the mass of the gas molecules, and *V* is the total available free space volume.

4. Simulation parameters

For simulating collision frequency, the nanostructure is defined as an imaginary box (Figure S11, and S12) and the laminar flow of gas molecules into the imaginary box is considered. The diameter of the nanofibers (*a*) is infinitely small compared to the size of the measurement chamber (*b*). Therefore, mimicking the size of the imaginary box is an important factor. As per the simulation results, when the imaginary box is as small as the inner diameter of the nanostructure, the ratio of the collision frequency of the inner surface to the outer surface is 1, indicating that there is no significant difference. When the ratio between the diameter of the nanofiber and the distance from the outer wall to the imaginary box (*b/a*) is greater than 30, the ratio of the collision frequency is saturated, indicating that the large size (*b/a* > 30) of the imaginary box is sufficient to simulate the real measurement situation.

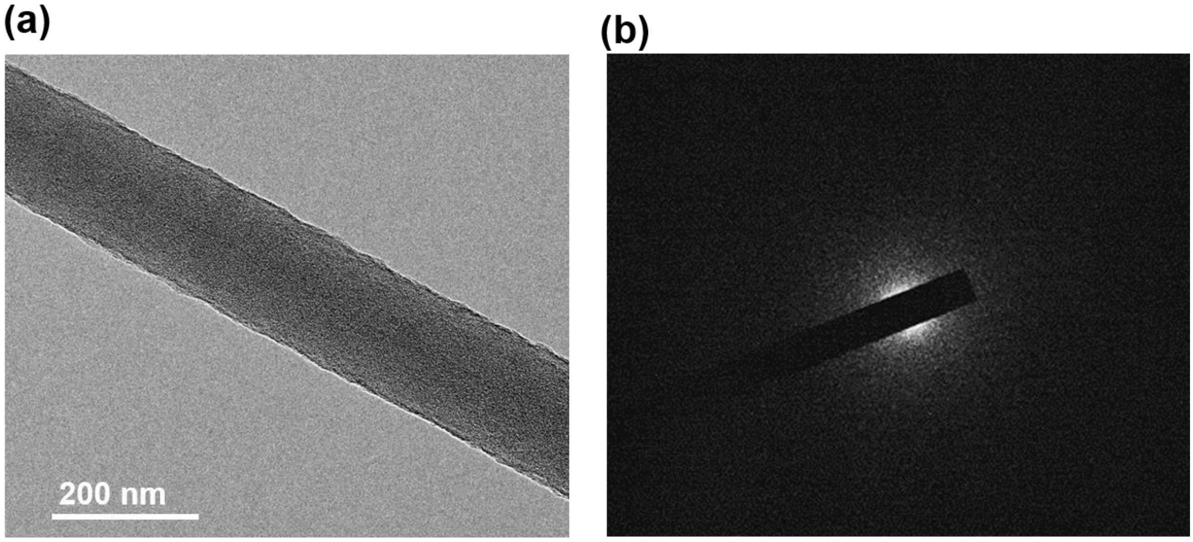

Figure S1. (a) TEM image, and (b) corresponding SAED pattern of the Al_2O_3 core fiber.

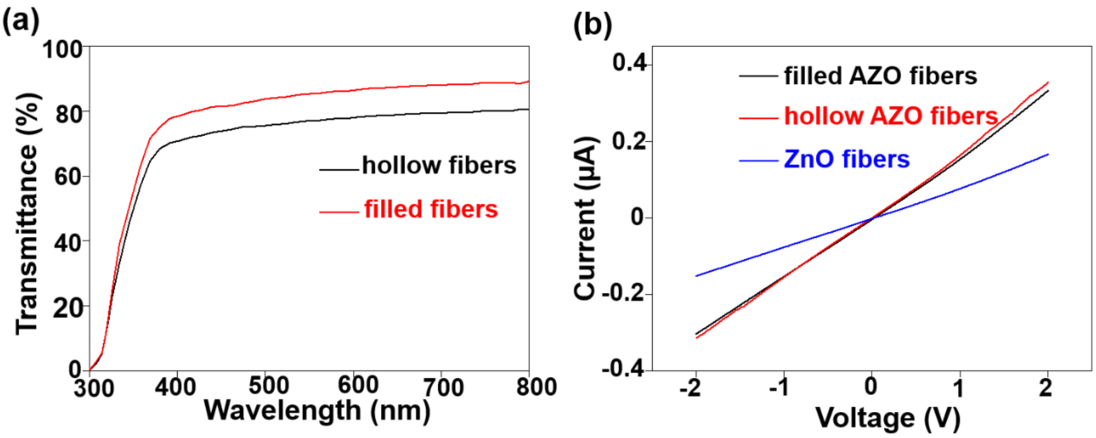

Figure S2. (a) UV-Vis spectra of free-standing hollow and filled AZO nanofibers, and (b) I-V characteristics curve of the free-standing hollow/filled AZO, and ZnO nanofibers.

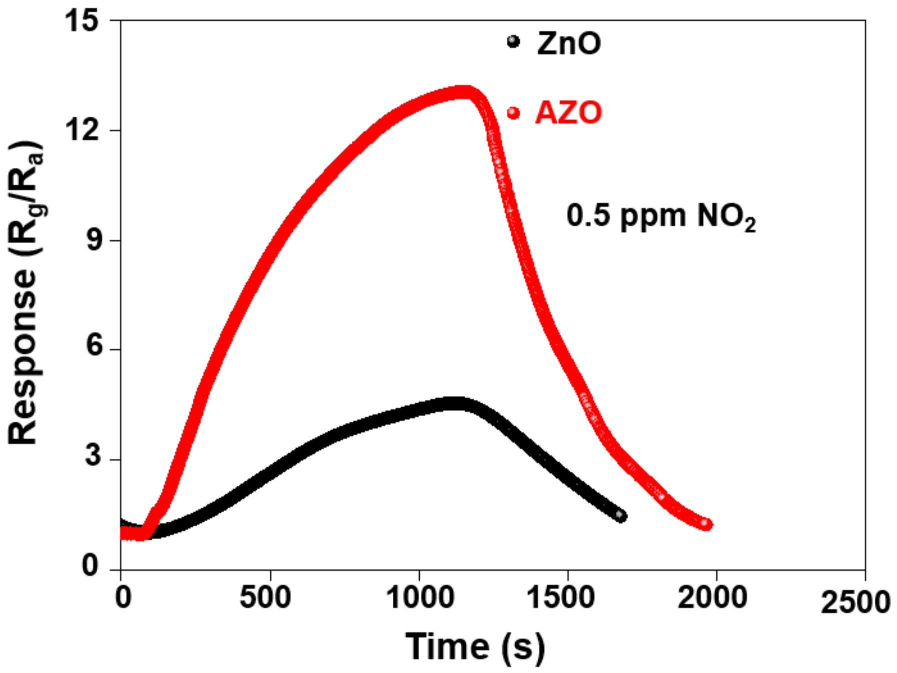

Figure S3. Comparative gas response curve of the free-standing AZO hollow nanofibers and ZnO nanofibers to 0.5 ppm NO_2 gas at 250°C.

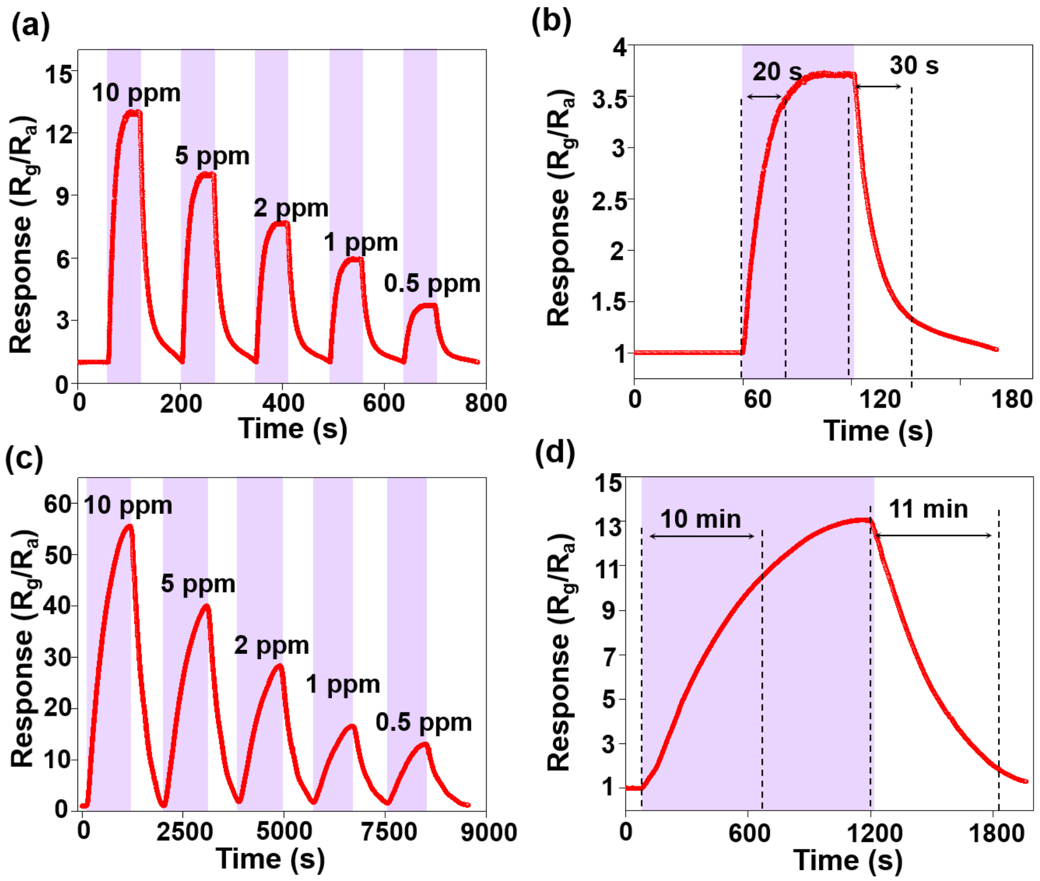

Figure S4. (a) Gas response curve of free-standing AZO filled nanofibers as a function of the NO_2 concentration (0.5-10 ppm) at 250°C, (b) gas response curve of free-standing AZO filled nanofibers as a function of time for 0.5 ppm NO_2 concentration at 250°C, (c) gas response curve of free-standing AZO hollow nanofibers for various NO_2 concentrations (0.5-10 ppm) at 250°C, and (d) gas response curve of free-standing AZO hollow nanofibers for 0.5 ppm NO_2 concentration at 250°C.

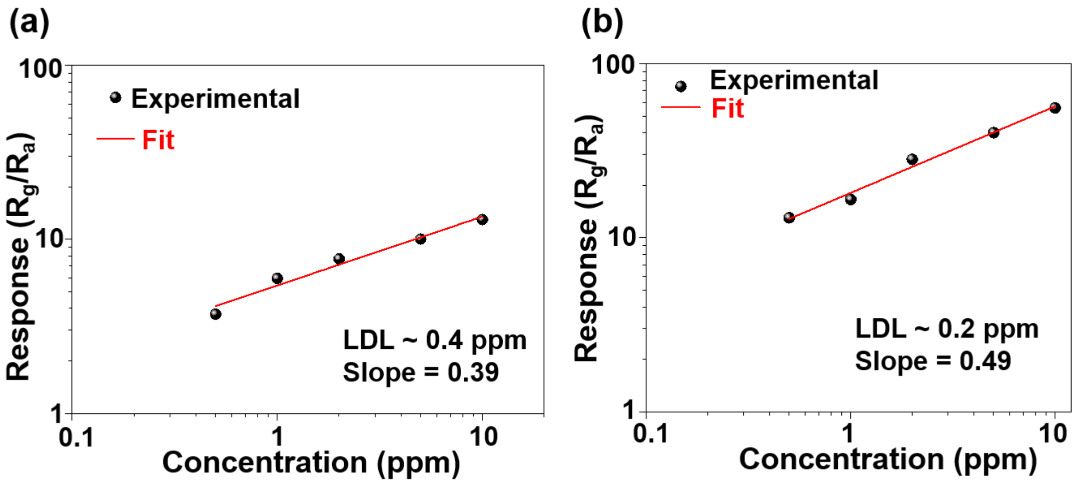

Figure S5. Linear fit of gas response versus concentration of NO_2 at 250°C for (a) filled and (b) hollow AZO nanofibers, respectively.

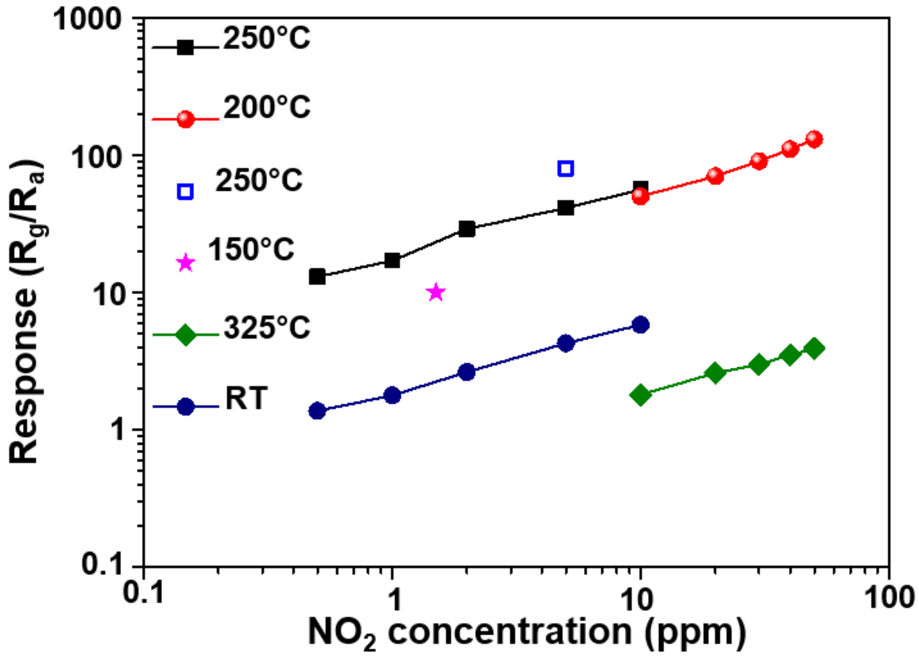

Figure S6. Gas-responses of our gas sensor as a function of NO_2 concentration at different operation temperatures in comparison with those of previously reported different types of gas sensors based on ZnO nanocrystals (●Ref. 33), In- and Sn-doped ZnO thin film (◘ Ref. 32, and ★Ref. 34) or ZnO nanowires (◆ Ref. 35).

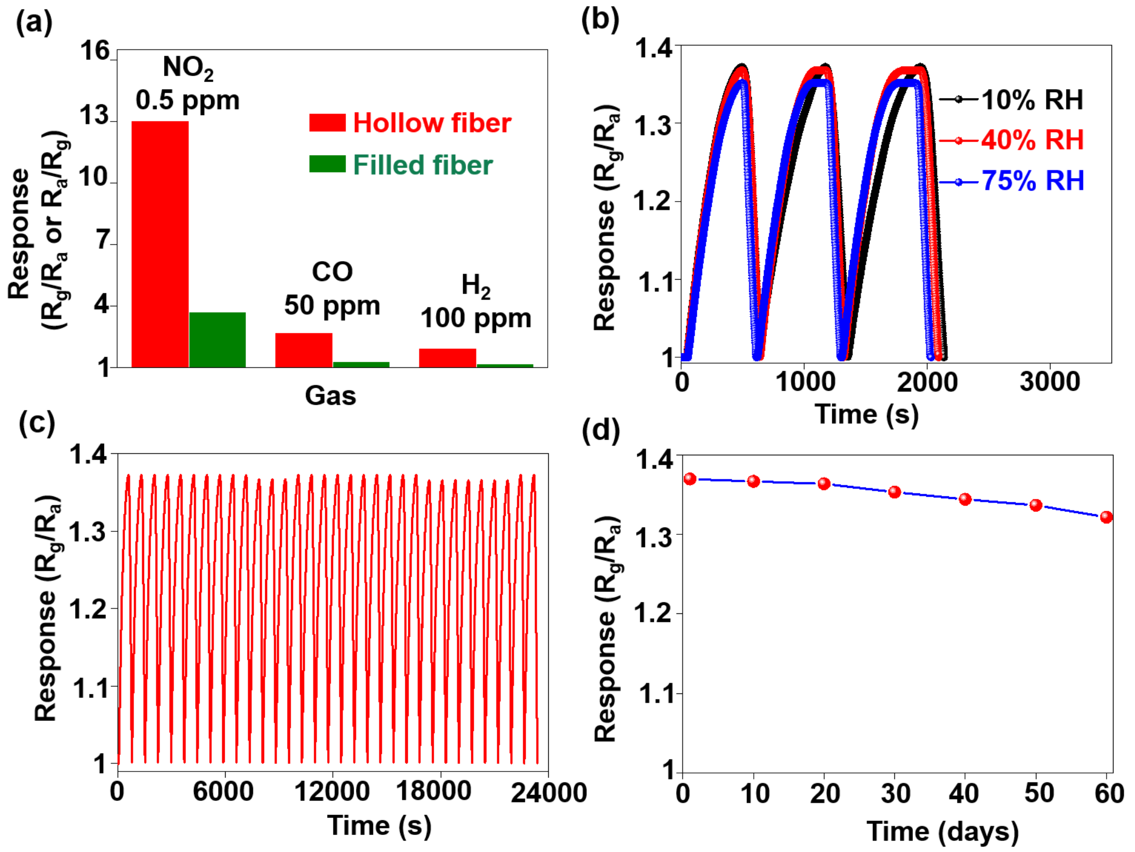

Figure S7. (a) Gas selectivity curves of the hollow and filled AZO fibers towards different gases with different concentration at 250 °C, (b) gas response curve of hollow AZO fibers towards 0.5 ppm NO_2 gas in different relative humidity conditions (10–75%) at RT, (c) cyclic stability curve of hollow AZO fibers towards 0.5 ppm NO_2 gas up to 30 cycles at RT, and (d) gas response retention behavior towards 0.5 ppm NO_2 for 60 days at RT.

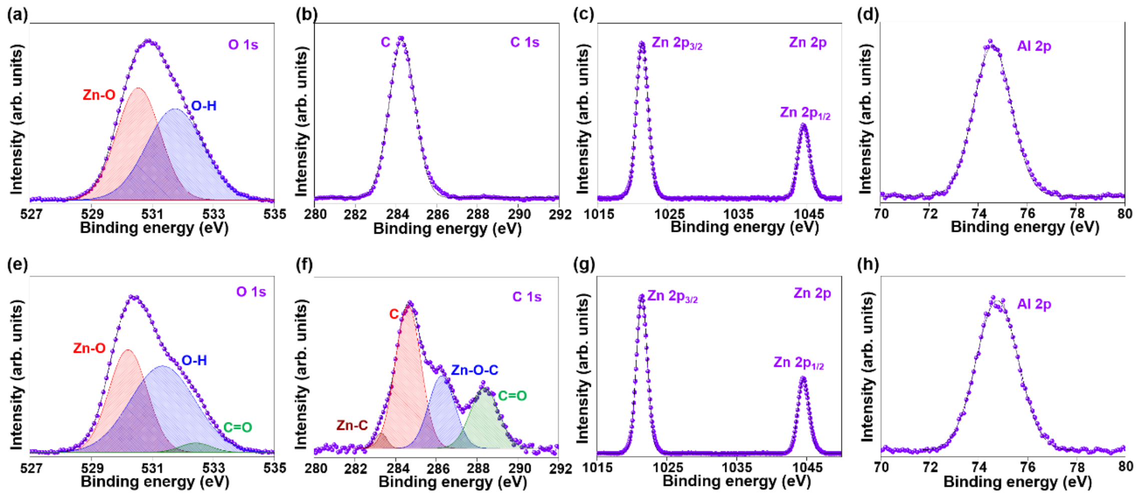

Figure S8. High resolution core level XPS spectra of AZO thin film (a) C1s, (b) O1s, (c) Zn2p, and (d) Al2p peaks. High resolution core level XPS spectra of hollow AZO fibers (a) C1s, (b) O1s, (c) Zn2p, and (d) Al2p peaks.

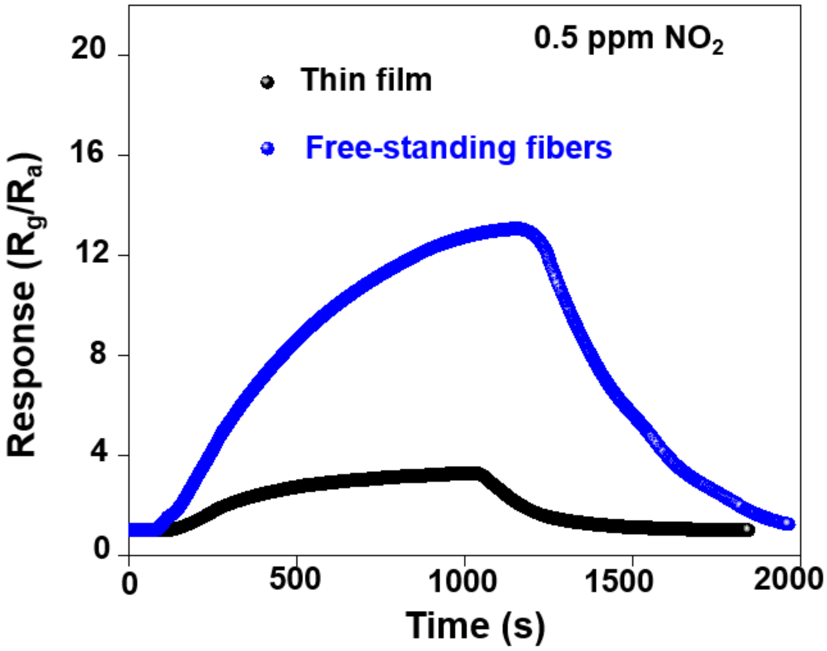

Figure S9. Comparative gas response curve of the free-standing AZO hollow nanofibers and thin film to 0.5 ppm NO_2 gas at 250°C.

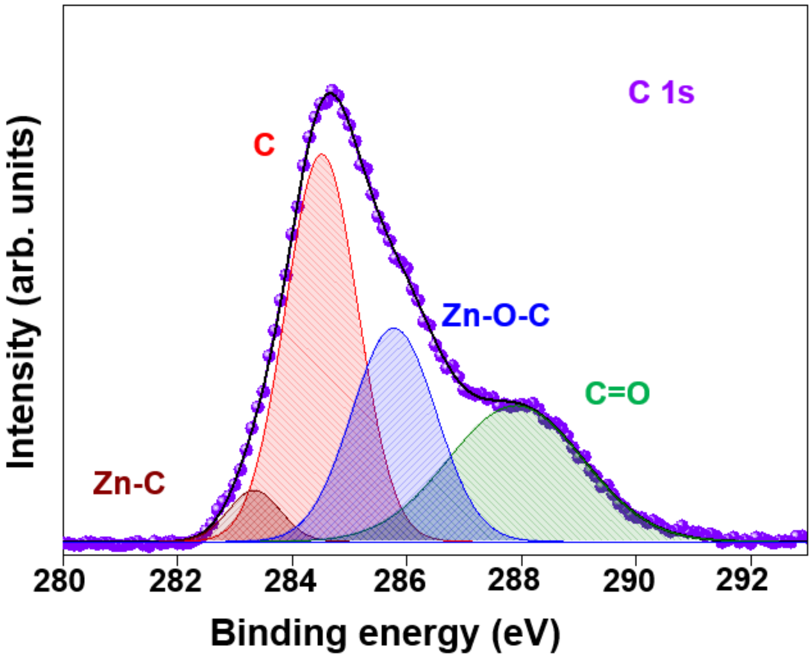

Figure S10. High resolution core level C1s XPS spectrum of filled AZO fibers.

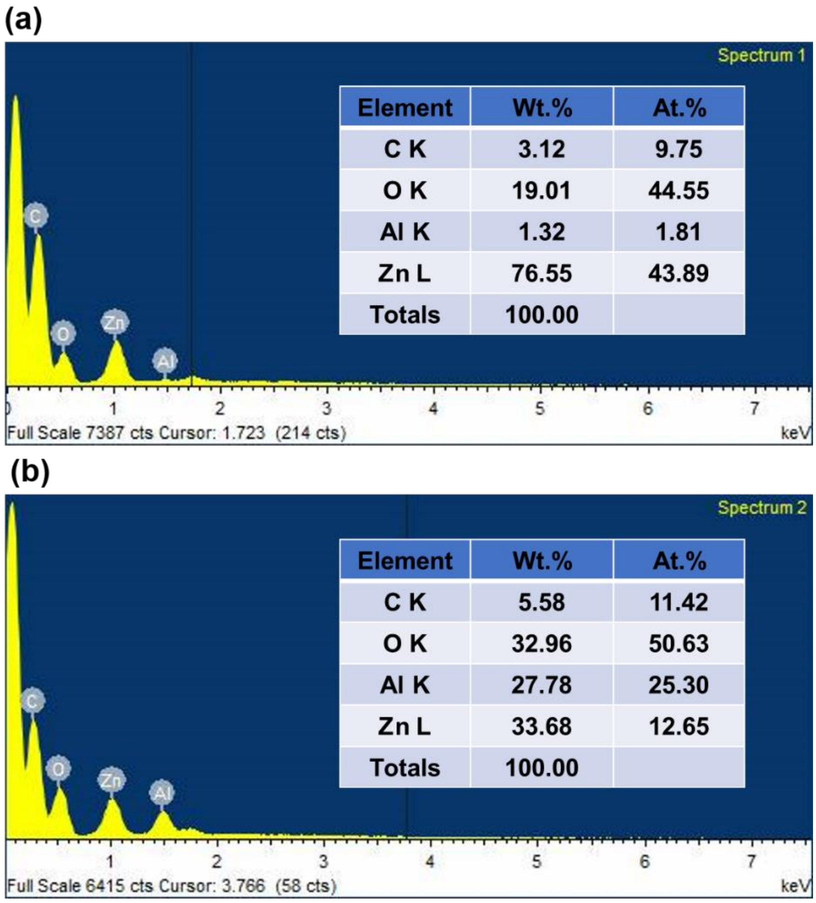

Figure S11. EDS spectra of (a) hollow, and (b) filled AZO fibers.

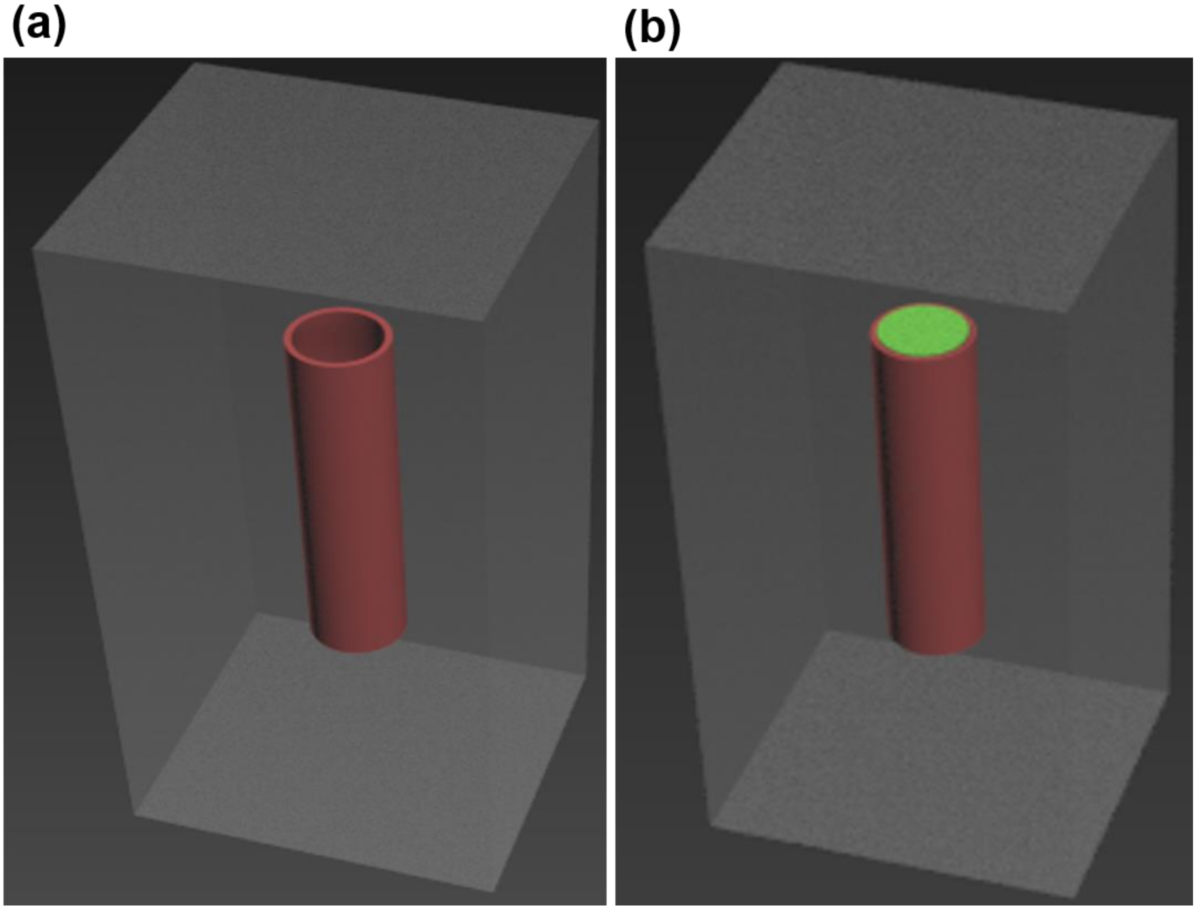

Figure S12. Schematic illustration of simulated active area for NO_2 gas sensing mechanism targeting (a) hollow, and (b) filled AZO nanofibers, respectively.

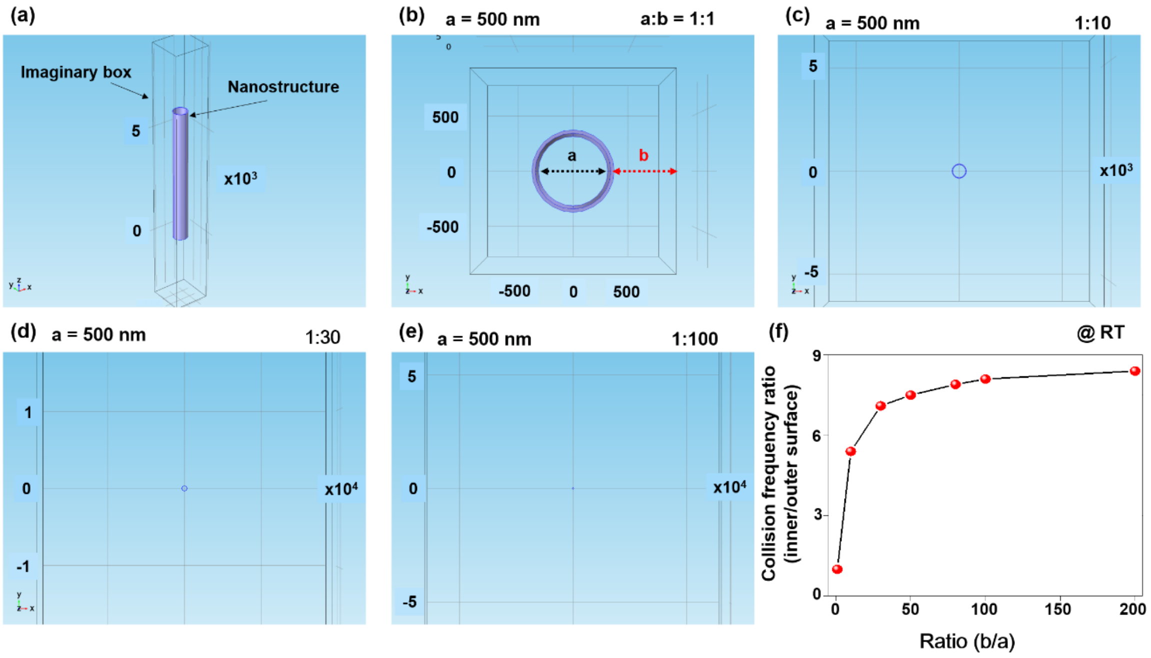

Figure S13. (a) Schematic illustration of simulated active area for nanofiber and imaginary box. The schematic graphs of different ratio between the diameter of the nanofiber a and the distance from the outer wall to the imaginary box b are shown in (b) a:b = 1:1, (c) a:b = 1:10, (d) a:b = 1:30, and (e) a:b = 1:100, respectively. (f) Graph between collision frequency ration in inner/outer surface vs. ratio between the diameter of the nanofiber a and the distance from the outer wall to the imaginary box b at room temperature.

References

1. O. Lupan, L. Chow, T. Pauporté, L. K. Ono, B. Roldan Cuenya and G. Chai, *Sensors and Actuators B: Chemical*, **2012**, *173*, 772.

2. V. V. Sysoev, J. Goschnick, T. Schneider, E. Strelcov and A. Kolmakov, *Nano Letters*, **2007**, *7*, 3182.

3. V. V. Sysoev, B. K. Button, K. Wepsiec, S. Dmitriev and A. Kolmakov, *Nano Letters*, **2006**, *6*, 1584.

4. O. Lupan, V. V. Ursaki, G. Chai, L. Chow, G. A. Emelchenko, I. M. Tiginyanu, A. N. Gruzintsev and A. N. Redkin, *Sensors and Actuators B: Chemical*, **2010**, *144*, 56.

5. O. Lupan, F. Schütt, V. Postica, D. Smazna, Y. K. Mishra and R. Adelung, *Scientific Reports*, **2017**, *7*, 14715.

6. O. Lupan, V. Cretu, V. Postica, M. Ahmadi, B. R. Cuenya, L. Chow, I. Tiginyanu, B. Viana, T. Pauporté and R. Adelung, *Sensors and Actuators B: Chemical*, **2016**, *223*, 893.
